# Agile Inverse Design of Polarization-Independent Multi-Functional Reconfiguration Metamaterials Based on Doped VO_2_

**DOI:** 10.3390/ma17143534

**Published:** 2024-07-17

**Authors:** Bingyao Shan, Yang Shen, Xuran Yi, Xianqing Chi, Kejian Chen

**Affiliations:** Shanghai Key Lab of Modern Optical System, Engineering Research Center of Optical Instrument and System, Ministry of Education, University of shanghai for Science and Technology, 516 Jungong Rd., Shanghai 200093, China

**Keywords:** metasurface, multi-functional, reconfiguration, electromagnetically induced absorption, vanadium dioxide, inverse design

## Abstract

Increasing attention is being paid to the application potential of multi-functional reconfigurable metamaterials in intelligent communication, sensor networks, homeland security, and other fields. A polarization-independent multi-functional reconfigurable metasurface based on doped vanadium dioxide (VO_2_) is proposed in this paper. It can be controlled to switch its function among three working modes: electromagnetically induced absorption (EIA), electromagnetically induced transparency (EIT), and asymmetrical absorption. In addition, deep learning tools have greatly accelerated the design of relevant devices. Such devices and the method proposed in this paper have important value in the field of intelligent reconfigurable metamaterials, communication, and sensing.

## 1. Introduction

With the development of advanced communication technologies and the continued evolution of non-contact sensing and related fields, the demand for intelligent control of electromagnetic waves has become increasingly pressing. Metamaterials are smartly engineered structures with rationally designed structures and are composed of periodic subwavelength metal/dielectric structures that resonantly couple to the electric and/or magnetic components of the incident electromagnetic fields, exhibiting special properties. They have wide applicability in filtering [1,2,3,4], metalenses [5], negative refraction [6], EIT [7,8,9], EIA [10,11,12], nonreciprocal transmission [13], and sensing [14,15]. Typically, a metasurface exhibits a specific functionality upon fabrication, and enhancing their versatility through conventional approaches can result in substantial augmentation of complexity and manufacturing expenses. The functional diversity and switching convenience of devices can be realized by empowering them with the ability of functional reconfiguration and nonlinear response [16], for example, by incorporating active media, such as phase change material and liquid crystals, and by using thermal effects [17] and ultrafast nonlinear optical effects [18] on conventional metasurfaces [19]. Phase change materials have a wide range of applications in microfluidics, dynamic wave modulation [20,21], and sensing. Vanadium dioxide (VO_2_) is a typical temperature-dependent phase change material. Sun et al. [22] proposed a reconfigurable broadband polarization conversion metasurface. Zhu et al. [23] designed a thermally controlled optical encryption metasurface using VO_2_. Wang et al. [24] proposed a transmission/reflection mode switchable ultra-broadband metasurface filter. Undoped VO_2_ exhibits a transition from an insulating dielectric state to a conducting metallic state at a critical temperature of approximately 67 °C (340 K) [25], while by doping tungsten ions in VO_2_ [26], the critical temperature of the phase transition can be reduced by 21~28 °C/at. % to about 42 °C (315 K) [27]. Thereby, multi-functional reconfigurable metasurface devices can be achieved by utilizing the VO_2_ materials with different temperature-controlled phase change points in diverse structures.

In this paper, a multi-functional reconfigurable metasurface that achieves EIA, EIT, and asymmetrical absorption by using VO_2_ materials in different structures is proposed. With the increasing complexity of devices, traditional structure-optimization methods are time-consuming and labor-intensive. To address this issue, we propose a deep learning-based inverse design method to accelerate the design optimization process and achieve favorable outcomes. The multi-functional reconfiguration metasurface and the related research methods proposed in this paper hold significant potential for applications in the design of intelligent metamaterials, as well as in communication and homeland security fields.

## 2. Structure Design and Simulation

**Figure 1:**
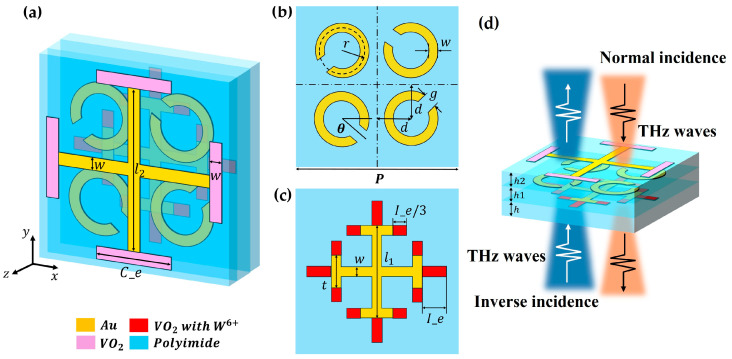
(**a**) The schematic of the proposed multi-functional reconfigurable metasurface unit. (**b**) The top view of the second layer (C-type SRRs). (**c**) The top view of the bottom layer (I-type cross and extend structures). (**d**) Side view of the device and two incidence directions.

The unit cell of the proposed multi-functional reconfigurable metasurface device is shown in Figure 1. Every part, from top to bottom, of the metasurface’s structure was a gold cross structure with a VO_2_ extender, gold C-type split-ring resonators (SRRs), and a gold I-type cross with extended structures of tungsten-ion-doped VO_2_. The thickness of the polyimide films, from bottom to top, are h, h1, and h2, respectively. The structural parameters were optimized after inverse design using the DNN model and are as follows: h = 10 μm, h1 = 15 μm, h2 = 8 μm, P = 160 μm, w = 10 μm, l1 = 78 μm, t = 20 μm, I_e = 36 μm, d = 30 μm, g = 10 μm, r = 18 μm, l2 = 118 μm, C_e = 63 μm. The thickness of the gold used and VO_2_ structures was set to 0.5 μm. The four C-type SRRs were placed in the four quadrants, respectively, and the angle to the adjacent axis was θ = 45°.

The electromagnetic responses of the device are effectively investigated by CST Microwave Studio. Unit cell boundary conditions are applied in the x and y directions, while an open boundary is set in the z direction. Gold is modeled as a lossy metal, and its conductivity is $4.561\times 10^{7}\;S/m$ [25]. The loss equation of polyimide films can be described as $\varepsilon_{d}=3.5+0.009i$. The dielectric permittivity of VO_2_ in the terahertz range can be characterized by the Drude model [28,29], expressed as $\varepsilon \left(\omega \right)={\;\varepsilon }_{\infty }-\omega_{p}^{2}\left({\;\sigma }_{1}\right)/{(\omega }^{2}+i\gamma \omega )$, where ${\;\varepsilon }_{\infty }$ is dielectric permittivity at the infinite frequency. Collision frequency γ is set to$\;\gamma =5.75\times 10^{13}\;rad/s,$ the plasma frequency$\;\omega_{p}$, which depends on σ, can be approximated by $\omega_{p}^{2}\left(\sigma \right)=\frac{\sigma }{\sigma_{0}}\omega_{p}^{2}\left(\sigma_{0}\right)$, with $\sigma_{0}=3\times 10^{5}\;S/m$ and $\omega_{p}\left(\sigma_{0}\right)=1.4\times 10^{5}\;rad/s$ [28]. In our simulation, both doped and undoped${\;\varepsilon }_{\infty }=12.$ VO_2_ is modeled as a material presenting a conductivity of 200 S/m in the insulating state, while in the metallic state, the conductivity is modeled as $2\times 10^{5}\;S/m.$

**Figure 2:**
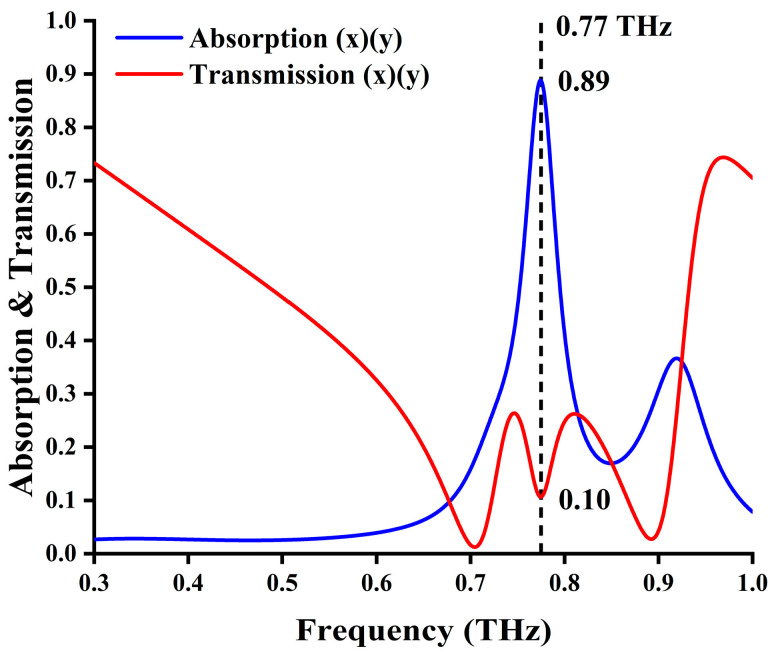
Absorption and transmission spectrum of the proposed multi-functional reconfigurable metasurface when the VO_2_ is in its insulated state at room temperature. The device is optimized by inverse design network.

Since the device structure has rotational symmetry around the device axis, under normal incidence, it demonstrates the same absorption characteristics for different polarizations, as shown in Figure 2; therefore, the device has polarization-independent characteristics.

## 3. Results and Discussion

### 3.1. Working States of Device Changes with Temperature

#### 3.1.1. (State 1)—EIA Phenomenon at Room Temperature

When the temperature is near room temperature (i.e., not exceeding 42 °C (315 K)), both doped and undoped VO_2_ is in an insulated state (200 S/m for both). In this condition, the device exhibits significant electromagnetic wave absorption characteristics, as shown in Figure 2. The absorption rate A of the device can be calculated using $A\left(\omega \right)=1-R\left(\omega \right)-T\left(\omega \right)=1-{\left|S_{11}\right|}^{2}-{\left|S_{21}\right|}^{2},\;$where$\;R\left(\omega \right)$, $T\left(\omega \right)$, $S_{11}\left(\omega \right)$, and $S_{21}\left(\omega \right)$ are the reflectance, transmittance, reflection coefficient, and transmission coefficient, respectively. The reflectance and transmission for forward (inverse) incidence are ${\left|S_{11}\right|}^{2}\left({\left|S_{22}\right|}^{2}\right)$ and ${\left|S_{21}\right|}^{2}\left({\left|S_{12}\right|}^{2}\right),$ respectively. As shown in Figure 2, the absorption peak is mainly caused by the coupling of three metal structural layers (more details will be discussed later); it also produces a transmission valley at 0.77 THz, which can further confirm the generation of EIA phenomenon. Its operating frequency is like the frequency of the resonance reflection of the single-layer structure and the EIT phenomenon of the two-layer metamaterial.

#### 3.1.2. (State 2)—Eit-like Phenomena When the W Ion-Doped VO_2_ Turns to the Metallic State

When the temperature rises into the range from 42 °C (315 K) to 67 °C (340 K), the VO_2_ doped with tungsten ions enters a metallic state while the undoped VO_2_ remain in an insulated state. In this condition, the working frequency of the third-layer I-type cross resonator jumps to 0.50 THz, and only the first-layer cross is coupled with the second-layer SRR. The coupling results in an EIT-like phenomenon.

**Figure 3:**
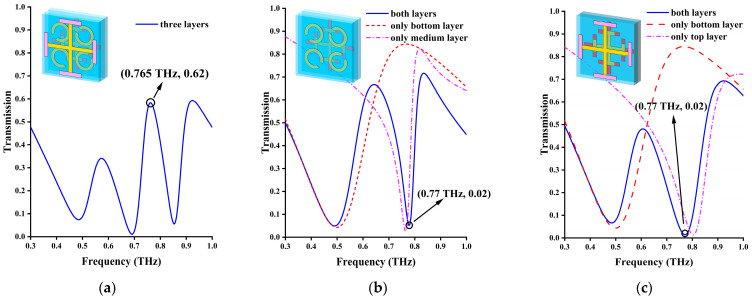
Doped VO_2_ is in the metallic state at temperature range of 42~67 °C. (**a**) EIT-like phenomenon of three layers of metamaterials acting together. (**b**) Transmission spectrum of the second layer SRR and I-type of the third layer. (**c**) Transmission spectrum of the first layer cross and I-type of the third layer.

Figure 3a shows the results of the transmission calculation under normal incidence. At this time, the three-layer device is divided into two functional modules: firstly, the devices that can produce an EIT-like effect at 0.765 THz and are formed by the interaction between the cross on the top cross layer and the SRR on the second layer and, secondly, a single-frequency filter formed by an I-type cross of the third layer at 0.46 THz, as shown in Figure 3b,c. At 0.765 THz, the transmittance can reach 0.62, while the transmittance at 0.46 THz is as low as 0.07. Figure 3b,c provides the proof that the EIT-like effect is mainly caused by the coupling of the top cross layer and the SRR layer. The EIT results are from a coherent cancellation between two different paths in a three-level atomic system. Through the EIT phenomenon, the transmission phenomenon can be generated in the same frequency of the initially non-transmitted structure.

In this device, when the incident wave occurs with x polarization, the cross acts as the bright mode, and the C-shaped SRR acts as the dark mode. The opposite is true when the incident wave occurs with y polarization. The coherent cancellation between the cross and the SRR ring results in this transition from reflection and absorption to transmission at the frequency of 0.77 THz.

#### 3.1.3. (State 3)—Asymmetrical Absorption at High Temperature

**Figure 4:**
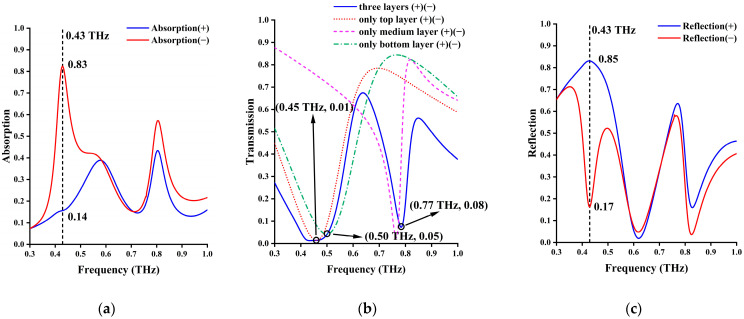
When both VO_2_ phases change into metallic state above 67 °C. (**a**) Absorptivity; (**b**) transmittance; (**c**) reflectivity.

In our design, when the temperature of the device is above 67 °C (340 K), both VO_2_ (undoped and doped) phases become metallic. As shown in Figure 4, changes in geometric parameters of the cross layer and the I-type cross layer due to VO_2_ metallization and incident terahertz waves can be coupled to the metamaterial structures of the first and third layers at 0.45 THz and 0.50 THz, respectively, while the second layer still maintains a resonance frequency of 0.77 THz. When the cross of the first layer interacts with the I-type cross of the third layer, an asymmetric absorption effect is generated at 0.43 THz, with a lower absorption rate (0.14) from the cross side (normal incidence) and an absorption rate of 0.82 from the I-type cross side (reverse direction). This phenomenon of asymmetric absorption has a certain application prospect in electromagnetic isolation and stealth. Meanwhile, at 0.765 THz, due to the resonance reflection effect of SRR, the transmittance of both incidence directions is suppressed below 0.08, and the reflectance reaches 0.64 in normal incidence and 0.55 in the reverse incident direction. Other energy, which is induced by residual coupling (not fully decoupled) between SRR and the primary metal structure of the first and third layers, is absorbed. If complete decoupling can be achieved, then the perfect resonance reflection can be achieved at this frequency point, while the proposed structure temporarily cannot achieve complete decoupling.

### 3.2. Discussion of the Working Mechanism and Other Related Issues

**Figure 5:**
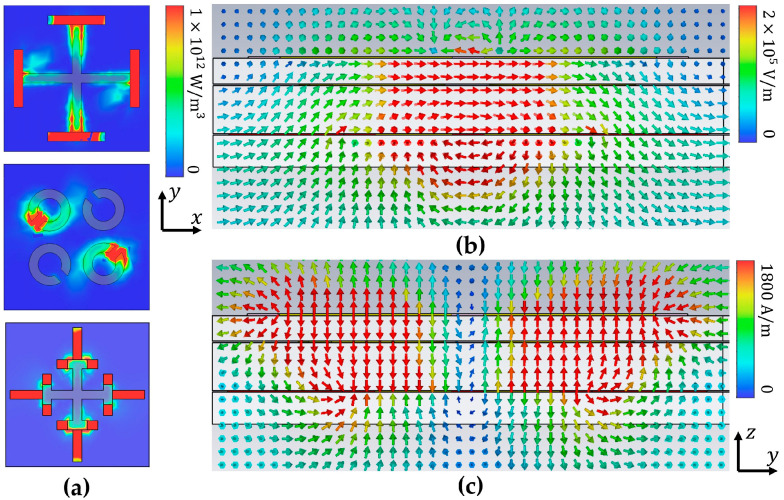
Simulation results of (**a**) power loss, (**b**) electric fields, and (**c**) magnetic fields at 0.77 THz, when they are at room temperature.

To better understand the phenomena and physical mechanisms that produce EIA phenomena at room temperature, the energy loss distribution of the proposed structure is analyzed, and the corresponding energy loss distribution when the incident wave in the x polarization direction is shown in Figure 5a. The electric and magnetic fields in the longitudinal section of the device are shown in Figure 5b,c. The EIA effect generated by this structure is mainly due to the vertical coupling between the three resonators. Due to the EIA effect, at 0.77 THz, most of the energy is lost through the SRR and the I-type cross, and a small part of the energy is lost through the cross-layer. The cross will generate strong loss points at the upper and lower two endpoints; the mechanism of the I-type cross is similar to the cross. The energy gathered in the metal will be lost in the VO_2_ and on the surface of the PI film due to the characteristics of the magnetic loss material without phase transition. The SRR ring shows a diagonal activation state due to the x polarization incident, and the energy is mainly lost at the gap between the SRRs and the PI dielectric material of the first and second layers.

**Figure 6:**
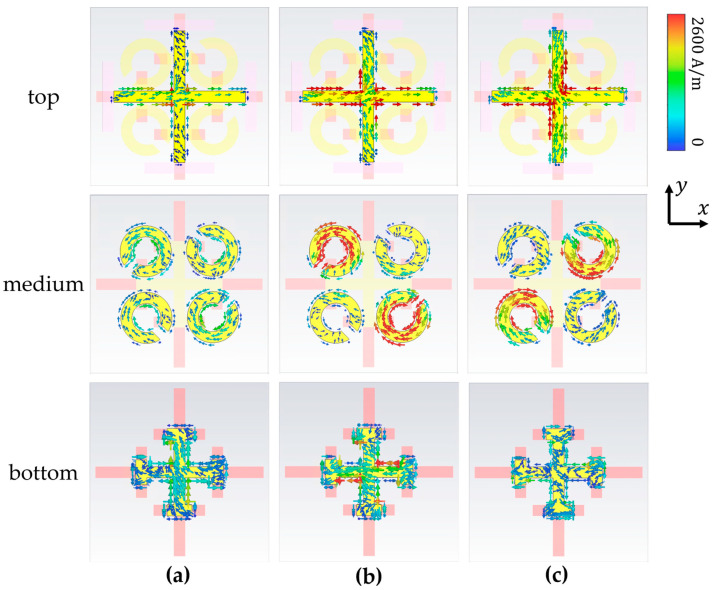
Surface current distribution of the three-layer structure at frequencies of 0.70 THz (**a**), 0.765 THz (**b**), and 0.855 THz (**c**) at the range of 42 °C (315 K) to 67 °C (340 K).

When the temperature rises into the range of 42 °C (315 K) to 67 °C (340 K), the bottom VO_2_ undergoes a phase change, and an EIT-like phenomenon occurs between the first and second layers. In order to better understand the physical properties of this EIT behavior, the surface current distribution in Figure 6 is calculated at the frequencies of two transmission troughs and one transmission peak, when the polarization direction of the incident wave is x polarization. Since the operating frequency of the bottom layer is moved to 0.43 THz under the phase-change action of doped VO_2_, the interaction of the three-layer structure at 0.77 THz becomes only a strong interaction between the first and second layers. Under the action of the external incident polarized wave, the bar parallel to the incident electric field is excited. Due to the interaction between the first and second layers, strong coupled resonance is excited on the second and fourth quadrant SRRs of the second layer (which cannot be directly strongly excited by the external × polarization incidence). As a result of the strong interaction between the first layer bar and the second SRR, electromagnetic-induced transmission occurs. Although the first and second layers also interact at frequencies of 0.70 THz and 0.855 THz, the current of the second-layer SRR induced by it is either very weak (such as 0.70 THz) or has a phase difference with the surface current of the first layer (opposite direction, @ 0.855 THz); thus, EIT cannot be achieved in these places. From the observed electric field distributions on the surfaces, it can be determined that the strong interaction between the cross of the first layer and the gold SRR of the second layer is the main reason for the electromagnetically induced transparency.

**Figure 7:**
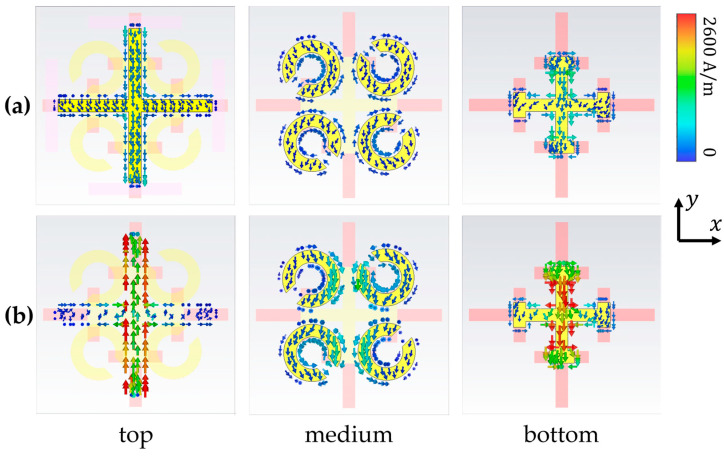
Surface current of the three-layer structure at 0.43 THz. (**a**) Forward incidence and (**b**) reverse incidence when temperature is above 67 °C (340 K).

When the temperature rises to above 67 °C (340 K), both doped VO_2_ and undoped VO_2_ become metallic. At this time, asymmetric absorption can be observed at 0.43 THz. The absorption is weak at forward incidence and very strong at reverse incidence. The absorption is mainly formed by the coupling of the first layer of the cross and the bottom layer of the I-shaped crosses with their VO_2_-extended structures. The operating frequency of the second layer of the SRR ring is fixed at 0.77 THz, so it has no effect on the absorption of this frequency. As shown in Figure 7a, at forward incidence, the current of the three-layer structure is small and has little effect on the incident wave. In Figure 7b, the strong absorption at reverse incidence is caused by the antiparallel current between the top cross and the bottom VO_2_ film. This current loop generates an artificial magnetic dipole moment which will interact strongly with the incident magnetic field and contribute to the absorption.

### 3.3. Potential Fabrication Process of the Proposed Structure

**Figure 8:**
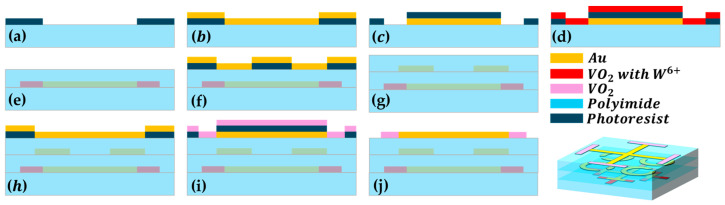
Potential fabrication process of the processed structure. (**a**) photolithography; (**b**) metal deposition; (**c**) liftoff and overlay photolithography; (**d**) doped-VO_2_ deposition; (**e**) liftoff and covered with polyimide layer; (**f**) the process for the second metal layer; (**g**) liftoff, and cover polyimide layer; (**h**) the process for the third metal layer; (**i**) liftoff, and deposit VO_2_ layer; (**j**) liftoff, and complete the device fabrication.

In practical applications, conventional microelectronic semiconductor process-related procedures can be considered for the fabrication of devices. These procedures include standard ultraviolet lithography [30] with thin-film deposition techniques [31]. The possible manufacturing process can start with a 10 μm PI film, as shown in Figure 8. The following instructions would need to be carried followed: (a) spin-coat photoresist and perform photolithography; (b) deposit a 0.5 μm thick metal layer on the PI film using magnetron sputtering or electron beam evaporation; (c) dissolve the photoresist, leave the gold pattern, spin-coat the photoresist again, and perform overlay process for photolithography; (d) magnetron sputter a 0.5 μm thick doped VO_2_ layer; (e) dissolve the photoresist and cover with a 15 μm thick polyimide layer; (f) spin-coat photoresist, perform photolithography, and then deposit the metal layer; (g) dissolve the photoresist and cover with an 8 μm thick polyimide layer again; (h) spin-coat photoresist, perform optical lithography, and deposit a 0.5 μm thick metal layer by electron beam evaporation; (i) after dissolving the photoresist, spin-coat the photoresist again, perform photolithography and magnetron sputtering of a 0.5 μm thick VO_2_ layer, and anneal the remaining VO_2_; (j) dissolve the photoresist to complete the device fabrication. Then, a THz-TDS system can be used for spectrum measurement. As the focus of this paper is to explore the application value of deep learning in the field of convenient reverse design of multifunctional reconfigurable metamaterials, those seeking more details regarding the possible fabrication and measurement method can refer to the papers mentioned above.

**Figure 9:**
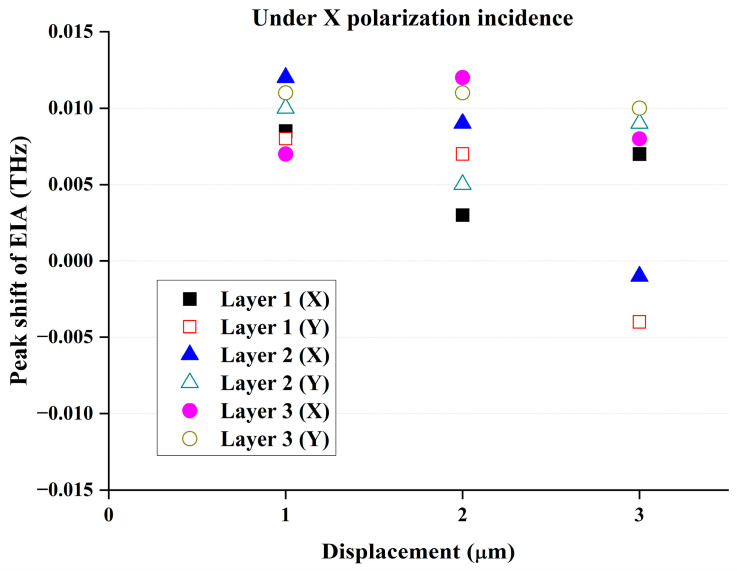
The effect of the displacement of metasurface layers (simulation results).

From the simulation results in Figure 9, it can be concluded that if the misplacement between the metasurface structure layers is within 3 μm, the spectrum of the devices will mostly have a slight blue shift, and a minor red shift may occur when the first layer shifts towards the y direction and the second layer shifts in the x direction. Because the precision of the photolithography-related technology is much better than that of 3 μm, the fabrication of the proposed device is realizable, although the process complexity may be relatively high.

### 3.4. Inverse Design Optimization Based on Deep Neural Network (DNN)

**Figure 10:**
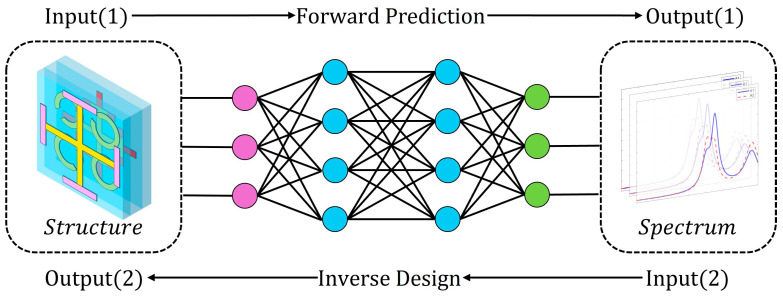
Flow chart of forward prediction and inverse design using DNN.

Machine learning (ML) offers a promising solution that can generate results with limited computing resources and reduce time-consuming computing processes [32]. The design process includes two steps: forward design and reverse design (see Figure 10). This paper selects a deep neural network (DNN) as the deep learning model. A DNN usually contains multiple hidden layers, which provide enough hidden units to represent complex functions according to the universal approximation theorem [33]. Therefore, it is possible to reveal the hidden relationship between variables. To train a DNN, several parameters of electromagnetic spectra and metasurfaces were defined as data sets. Because deep learning is used for the agile design of the device, the absorption spectrum in the x polarization direction of the polarization-independent metamaterial structures is used for data collection and model training. During the data collection process, structural parameters were generated randomly to reduce the difficulty of training. Five structural parameters that have a significant impact on the spectral response (for example, EIA) were selected for data collection. The parameters and their ranges are shown in Table 1.

The parameter table generated was saved into a file, and the spectral line data in the front and rear incident directions of the device were collected using Matlab-CST joint simulation. A total of 2592 sets of valid data were simulated. Then, the entire data set was divided as follows: 80% into a training set and 20% into a test set.

**Figure 11:**
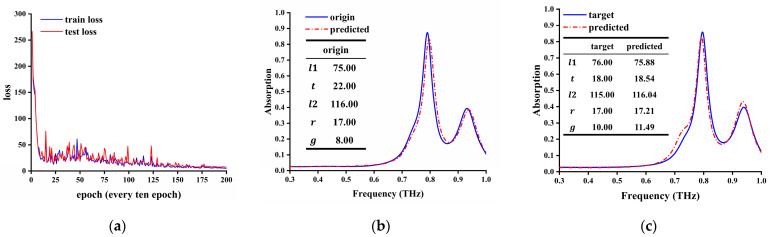
(**a**) The loss curve of training and test. (**b**) Forward design and (**c**) inverse design of target and predicted spectrum of structures.

Here, we use the EIA state of three-layer coupling as an example to illustrate how to inverse design a more complex multi-function metamaterial device. The data set collected from the previous simulation was entered into the model for training. After 2000 iterations, when the test loss of the test set drops to 4.7, the training process converges, and the loss becomes stable. The loss curve of the test set is shown in Figure 11a. Once the inverse learning model is trained, we can predict the desired structural parameters through the generator in a few milliseconds.

To further verify the validity of model-based prediction parameters, spectra were predicted by the forward network (Figure 11b). The predicted device structure parameters generated by the inverse network were rounded and simulated again in CST 2022 software, and the predicted spectra were compared with the target spectra (Figure 11c). The blue solid line is the target simulation curve, and the red dashed line is the simulation result of the DNN predicting structural parameters.

It can be seen from the results that the spectra obtained by our prediction model can accurately describe the EIA effect generated by the three-layer structure, and the results also show that a DNN has good learning ability and adaptability and can be extended to the design of new metasurfaces.

**Figure 12:**
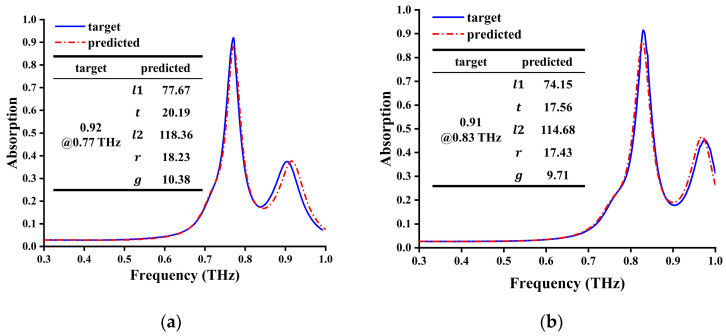
The ideal EIA spectrum and simulation result at (**a**) 0.77 THz and (**b**) 0.83THz.

To achieve better EIA effect, we manually generated a spectrum with ideal absorption at two target frequencies (0.77 THz and 0.83 THz) and input them into the DNN model for inverse design and prediction of parameters. Then, we used CST software to simulate and verify it again. The obtained simulation results with the prediction of parameters are shown in Figure 12a,b with the ideal spectrum. The analysis shows that after using the DNN model for inverse design, the three-layer structure can be designed and optimized quickly and accurately. Due to the imperfection of the current model, the result may not be the optimal state of the structure. We can manually fine-tune the model parameters to achieve the best state based on the model parameters generated by the DNN model. By using inverse learning, the design time of complex devices can be greatly shortened to about 1/1000 to 1/10,000 of the original time.

## 4. Summary

A polarization-independent multi-functional reconfigurable metasurface is proposed. At room temperature, the proposed metasurface device exhibits the characteristics of EIA at 0.77 THz, and the absorption rate can reach 0.89 (Q factor is 14.81). When the doped VO_2_ is in the metallic state at a temperature range of 42~67 °C, the interaction between the top cross layer and the SRR layer results in an EIT-like phenomenon at 0.77 THz (its Q factor is 8.47), when the narrow-band filter at 0.5 THz. When the temperature keeps increasing and both VO_2_ (doped and undoped) are in the metallic state, asymmetrical absorption can be achieved at 0.43 THz, and the absorption rate can reach 0.82 (its Q factor is only 3.69, due to a flat stage); the absorption rate difference can reach 0.68. If needed, metasurface devices with higher Q value can be obtained by adopting some quasi-BIC resonance structures into the design (for example, asymmetric SRRs [3,4]), but the polarization-independent properties may be sacrificed. By leveraging the phase transition property of VO_2_, the proposed metamaterials can reconfigure the functionality of VO_2_ without altering its geometric shape. Simultaneously, the design optimization process was accelerated with a DNN and the inverse learning method to expedite the design optimization process. Consequently, VO_2_ can potentially revolutionize the development of optical switchable components, enabling multifunctionality within a single device, and the optimization method based on deep learning holds significant application value and broad prospects in intelligent metamaterial design, communication, and other related fields.

## Figures and Tables

**Table 1:** Scanning parameters.

Parameters	***l*** **1**	***t***	***l*** **2**	***r***	***d***
Min size (μm)	74	17	113	17	8
Max size (μm)	84	22	123	20	12
Samples	6	6	6	4	3

## Data Availability

The original contributions presented in the study are included in the article, further inquiries can be directed to the corresponding author.
